# Predictors of Immune-Related Cardiac Adverse Events in Patients Receiving Immune Checkpoint Inhibitors: A Retrospective Cohort Study

**DOI:** 10.3390/jcm15051763

**Published:** 2026-02-26

**Authors:** Ileana-Raluca Pătru, Andreea-Iuliana Ionescu, Alina Gabriela Negru, Alexandra-Valentina Anghel, Maria Iordache, Oana Georgiana Becheru, Ilinca Arina Baranescu, Ioana Livia Barascu, Dimitrie Ionut Atasiei, Ionuț-Lucian Antone-Iordache

**Affiliations:** 1 Medical Oncology Department, Colțea Clinical Hospital, 030167 Bucharest, Romania; ileana-raluca.patru@drd.umfcd.ro (I.-R.P.); maria.malai@drd.umfcd.ro (M.I.); 2 Faculty of Medicine, “Carol Davila” University of Medicine and Pharmacy, 020021 Bucharest, Romania; alexandra-valentina.anghel@stud.umfcd.ro (A.-V.A.); oana-georgiana.becheru0720@stud.umfcd.ro (O.G.B.); ilinca-arina.baranescu0720@stud.umfcd.ro (I.A.B.); ioana-livia.barascu0720@stud.umfcd.ro (I.L.B.); dimitrie-ionut.atasiei25@rez.umfcd.ro (D.I.A.); ionut-lucian.antone-iordache@drd.umfcd.ro (I.-L.A.-I.); 3 Radiotherapy Department, Colțea Clinical Hospital, 030167 Bucharest, Romania; 4 Cardiology Department, University of Medicine and Pharmacy “Victor Babes” Timisoara, 300041 Timisoara, Romania; alinanegru@umft.ro; 5 Neurosurgery Department, Emergency Clinical Hospital “Sf. Pantelimon”, 021659 Bucharest, Romania

**Keywords:** immune checkpoint inhibitors, immune-related adverse events, cardiotoxicity, cardiovascular comorbidities, immunotherapy, predictive factors, oncology, cardiac toxicity

## Abstract

**Background/Objectives**: Immune checkpoint inhibitors (ICIs) are associated with immune-related adverse events (irAEs), including cardiac toxicities, which may have significant clinical impact. Data regarding predictors of immune-mediated cardiac toxicity remain limited. The aim of this study was to evaluate the association between cardiovascular comorbidities and the development of cardiac irAEs, as well as to assess their potential role in predicting non-cardiac irAEs. **Methods**: We conducted a retrospective observational study including oncological patients treated with immune checkpoint inhibitors at Colțea Clinical Hospital. Patients with complete clinical data and documented cardiac and non-cardiac irAEs were included. Demographic characteristics, prior oncological treatments, cardiovascular comorbidities, and relevant clinical parameters were analyzed. Univariate and multivariate logistic regression analyses were performed to identify predictors of immune-related adverse events. **Results**: Hypercholesterolemia, atrial fibrillation, history of acute coronary syndrome, and heart failure were significantly associated with the development of cardiac irAEs. In multivariate analysis, hypercholesterolemia and pre-existing cardiovascular disease remained independent predictors of cardiac toxicity. No significant association was observed between non-cardiac irAEs and cardiac irAEs. The predictive model demonstrated high specificity but moderate sensitivity. **Conclusions**: Cardiovascular comorbidities play a significant role in the development of immune-related cardiac toxicities. Identification of these risk factors may improve patient stratification and support individualized monitoring strategies in patients receiving immune checkpoint inhibitors.

## 1. Introduction

Immune checkpoint inhibitors (ICIs) have profoundly transformed the therapeutic landscape of oncology and currently represent a cornerstone in the treatment of multiple solid and hematological malignancies. By blocking inhibitory immune pathways such as programmed cell death protein 1 (PD-1), its ligand PD-L1, and cytotoxic T-lymphocyte-associated antigen 4 (CTLA-4), these agents restore antitumor immune surveillance and significantly improve survival outcomes across a wide range of cancer types [1,2].

However, immune activation induced by ICIs is associated with the development of immune-related adverse events (irAEs), which may affect virtually any organ system. While many irAEs are mild and manageable, severe toxicities can occur and may necessitate treatment interruption or immunosuppressive therapy. Accumulating evidence suggests that the occurrence of irAEs is associated with improved oncologic outcomes, supporting the concept that immune activation underlies both therapeutic efficacy and immune-mediated toxicity [3,4]. Nevertheless, the mechanisms responsible for irAE development and their clinical predictors remain incompletely understood.

Among all irAEs, cardiovascular toxicities represent a particularly concerning subgroup due to their severity and high associated mortality, despite a relatively low incidence. Immune-related cardiac adverse events include myocarditis, pericarditis, arrhythmias, heart failure, and acute coronary syndromes, often characterized by early onset and an unpredictable clinical course [5,6]. Although uncommon, immune-related cardiotoxicity, especially myocarditis, is associated with disproportionately high fatality rates compared with other irAEs, underscoring the need for improved risk stratification and early identification of vulnerable patients.

To date, data regarding predictors of immune-related cardiac toxicity remains limited and heterogeneous. There is currently no definitive prediction model for cardiovascular irAEs [7]. In particular, the role of pre-existing cardiovascular disease and traditional cardiovascular risk factors in determining cardiac irAEs remains insufficiently defined. Furthermore, it is unclear whether cardiac irAEs share common biological mechanisms with non-cardiac irAEs or represent a distinct clinical entity driven by different pathophysiological pathways [8,9].

In this context, the primary objective of the present study was to determine whether specific cardiovascular conditions and comorbidities are associated with the development of immune-related cardiac toxicities in patients treated with immune checkpoint inhibitors. As a secondary objective, we aimed to investigate whether these same factors predict non-cardiac immune-related adverse events, thereby contributing to improved risk stratification and personalized monitoring strategies.

## 2. Materials and Methods

### 2.1. Study Design and Patient Population

We conducted a retrospective observational study of a cohort of patients diagnosed with various cancers and treated with immune checkpoint inhibitors (ICIs) at the Oncology Department of Colțea Clinical Hospital, Bucharest. The initial cohort was previously published by our research group [10], and the analysis presented in this article represents an extension and further exploration of the original findings.

**Figure 1:**
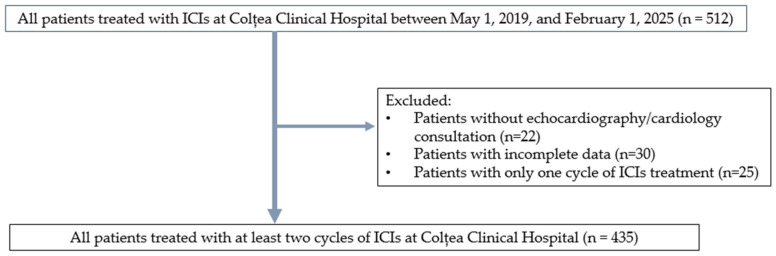
Overall study design. Reproduced from Pătru et al. [10].

As previously mentioned in Pătru et al. [10], inclusion criteria were adult patients (≥18 years) diagnosed with solid tumors and treated with ICIs in the Oncology Department of Colțea Clinical Hospital, Bucharest, Romania. Patients were excluded based on the absence of data concerning follow-up, survival, or adverse events; no cardiologic monitoring mentioned in the electronic records (no echocardiography or electrocardiogram before and during therapy); and fewer than two cycles of ICIs. The flow diagram of our cohort is presented in Figure 1.

### 2.2. Data Collection

In our previously published cohort, we collected variables regarding demographic data, oncological history, prior treatment exposure, cardiovascular comorbidities, relevant biological parameters, type of ICI administered, duration of ICI treatment (follow-up), immune-related adverse events, and survival data. IrAEs were categorized as cardiac and non-cardiac irAEs.

Cardiac irAEs were graded according to CTCAE v5.0 and included adverse events pertaining to myocarditis, pericardial disease (pericarditis or pericardial effusion), and arrhythmias (atrial fibrillation, conduction disturbances). Cardiovascular events not associated with ICI therapy were categorized as cardiomyopathy, vascular toxicity, and hypertension.

Our institutional protocol requires an ECG before each ICI cycle. Any new onset symptoms such as edema and dyspnea or chest pain require an additional ECG and referral to our in-hospital cardiologists that recommend further investigation (such as troponin, CK-MB and echocardiography). A joint oncologist–cardiologist team assesses each case, decides further management, and mentions the final diagnostic in the electronical record.

Non-cardiac irAEs were categorized as dermatologic (rash, pruritus, vitiligo), gastro-intestinal (colitis, diarrhea, hepatitis), endocrine (thyroid dysfunction, hypophysitis, adrenal insufficiency), pulmonary (pneumonitis), and renal (nephritis) toxicities.

### 2.3. Variables Studied

For the prediction of cardiac and non-cardiac irAEs, the following categories of variables were analyzed:

- General predictors: age, prior radiotherapy, prior chemotherapy, and previous treatment with anthracyclines (noting that the number of exposed patients was very limited).
- Cardiovascular comorbidities prior to ICI therapy: diabetes mellitus, chronic kidney disease*, hypercholesterolemia, arterial hypertension, history of acute coronary syndrome*, atrial fibrillation*, heart failure, and left ventricular ejection fraction*. Variables marked with ‘*’ were extracted for the current article and were not previously published.
- Immune-related events: presence of non-cardiac irAEs, evaluated as potential predictors for the development of cardiac irAEs.

All variables were extracted from official clinical records, interdisciplinary consultations, and investigations performed during the course of oncological treatment.

### 2.4. Statistical Analysis

We used JASP 0.95.1, R version 4.5.2 [11], and RStudio 2026.1.0.392 [12] for statistical analysis.

Nominal data were described as numbers and percentages; continuous data were presented as mean and standard deviation.

We performed univariate logistical regressions in order to see which general or cardiac-related variables can predict the appearance of cardiac irAEs; then, we selected the statistically significant predictors and included them in a multivariate logistic regression model. We checked overall model fit using McFadden R^2^ and discriminatory power using a Receiver Operating Characteristic (ROC) curve, calculating the area under the curve (AUC), sensitivity, specificity and Brier score. We used the rms package and val.prob [13] function to generate a calibration plot and the rmda package [14] to generate a decision curve analysis. We used the EValue library [15] and the evalues.OR function to calculate the e-value for unmeasured confounding variables as established by VanderWeele et al. [16]. Missing data was handled by complete-case exclusion from the simple and multiple logistic regression.

We used the same variables in order to predict the occurrence of non-cardiac irAEs; however, there were not enough significant predictors in order to build a multivariate model. After we performed this set of analyses on the general cohort, we wanted to see if the same variables can predict cardiac irAEs in our two most populous subgroups: patients with lung cancer and head and neck cancer.

Collinearity for the multivariate model was assessed using tolerance and variance inflation factor (VIF), with thresholds of >0.1 and <10 considered acceptable, respectively.

A *p*-value of 0.05 was considered statistically significant.

### 2.5. Ethical Considerations

This study was conducted in accordance with the ethical principles of the Declaration of Helsinki. As the analysis was performed retrospectively on anonymized data, without any intervention involving patients, ethical approval was handled in accordance with the internal procedures of the institution. All data were used exclusively for scientific purposes.

### 2.6. Limitations

Several limitations of the present study should be acknowledged. First, the retrospective and observational design inherently limits the ability to infer causal relationships between cardiovascular comorbidities and the development of immune-related cardiac adverse events (irAEs). Data collection relied on existing medical records, which may be subject to incomplete documentation and information bias, particularly regarding cardiovascular history and subclinical cardiac abnormalities.

Second, although the overall cohort size was acceptable, the number of patients included in subgroup analyses—particularly those with lung cancer and head and neck cancer—was relatively small. This limited statistical power may have contributed to the variability observed in predictor significance across subgroups and restricts the generalizability of these findings to specific oncological populations.

Third, the predictive model was developed and tested within the same cohort, which introduces the risk of optimism bias. Although the model demonstrated high specificity, its sensitivity was only moderate, indicating that a substantial proportion of cardiac irAEs cannot be fully explained by the variables included in the analysis. Consequently, external validation in larger, independent cohorts is required to confirm the robustness, reproducibility, and clinical applicability of the model, as well as to evaluate its performance across different cancer types and treatment settings.

Another important limitation is the absence of biological and inflammatory biomarkers, such as cardiac troponins, natriuretic peptides, or inflammatory cytokines, which could have provided additional insight into the underlying mechanisms of immune-related cardiotoxicity. In addition, the number of patients previously exposed to anthracyclines was very small, preventing a reliable assessment of their contribution to cardiac risk.

The heterogeneity of the study population, encompassing multiple tumor types and treatment regimens, limits the extrapolation of results to more homogeneous clinical settings. Furthermore, this study did not evaluate the temporal dynamics or long-term evolution of cardiac irAEs.

Finally, although mechanistic hypotheses regarding the roles of cardiovascular vulnerability and immune-mediated injury are discussed, the observational nature of this study does not permit direct investigation of causal biological pathways. Prospective studies integrating clinical, imaging, and biomarker data are needed to better elucidate the mechanisms underlying immune-related cardiac toxicity.

## 3. Results

### 3.1. Descriptive Data

The current extended analysis was performed on a cohort of patients that was previously published by our team in the article ‘The Impact of Immune-Related Adverse Events on the Survival of Patients Treated with Immune Checkpoint Inhibitors: The Distinct Role of Cardiac Toxicities’. The original cohort descriptives are represented in Table A1 and Table A2. The latter includes, among other information, the type of ICIs administered and cancer type; the former includes the grading for cardiac irAEs. There were 435 patients, of whom 55 patients developed documented cardiac irAEs. Median follow-up was 8.7 months after initiation of ICI therapy with a mean follow-up of 14.2 months (standard deviation of 15 months). We collected additional data on chronic kidney disease, atrial fibrillation, acute coronary syndrome, ejection fraction (FEVS) and heart failure prior to ICI therapy (Table 1). FEVS had a mean value of 56.5% (standard deviation of 5.05%).

### 3.2. Logistic Regression Models Predicting Cardiac irAEs for the General Population

In order to predict cardiac irAEs in our general population, we devised a model assessing both cardiac-related and non-cardiac related variables. Non-cardiac related variables were age, radiation therapy and chemotherapy prior to ICI. We wanted to assess if previous anthracycline treatment is a risk factor for cardiac irAEs, but only seven people in our cohort had been on this therapeutic agent. Another pre-specified predictor was non-cardiac irAEs. As shown in Table 2 none of them were statistically significant.

We assessed common cardiologic conditions and cardiac disease risk factors that patients had in their charts prior to immunotherapy through simple logistic regressions models. When evaluated in this way, a history of hypercholesterolemia, hypertension, acute coronary syndrome, atrial fibrillation and heart failure were risk factors for cardiac irAEs (Table 2).

We then introduced all the statistically significant predictors into a multiple logistic regression. Due to being close to achieving statistical significance, we also added age in the model to adjust for it. In this setting, age was clearly not significant, and hypertension was not significant anymore, suggesting that its effects on cardiac irAEs are co-linked with the other cardiologic conditions (Table 3).

Even though the model fit was not excellent, suggesting there are other factors not assessed by this study that influence the appearance of cardiac irAEs, the discriminatory power was acceptable.

**Figure 2:**
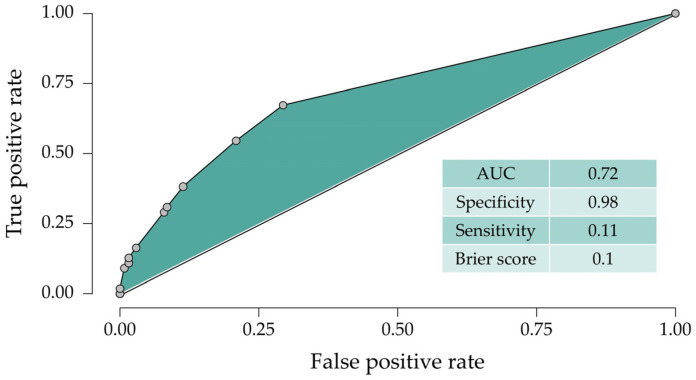
ROC curve of the model presented in Table 3. Area under the curve (AUC), specificity, sensitivity and Brier score were calculated.

We performed a ROC curve (Figure 2) and calculated the AUC, specificity, sensitivity and Brier score. The model proved to be highly specific, signaling that people with no cardiac disease pre-ICI therapy are very unlikely to develop cardiac irAEs. However, sensitivity indicates that our model does not fully explain the occurrence of cardiac irAEs. A Brier score this low indicates high accuracy, but this finding must be interpreted with caution, as this model could suffer from optimism bias. We did not include confounders regarding ICI regimen, line of therapy, concurrent systemic treatments, or cardiovascular disease medication; all of these confounders could explain the low model fit and low sensitivity, as these variables could be potential targets for bigger cohorts. Further external validation is needed.

**Figure 3:**
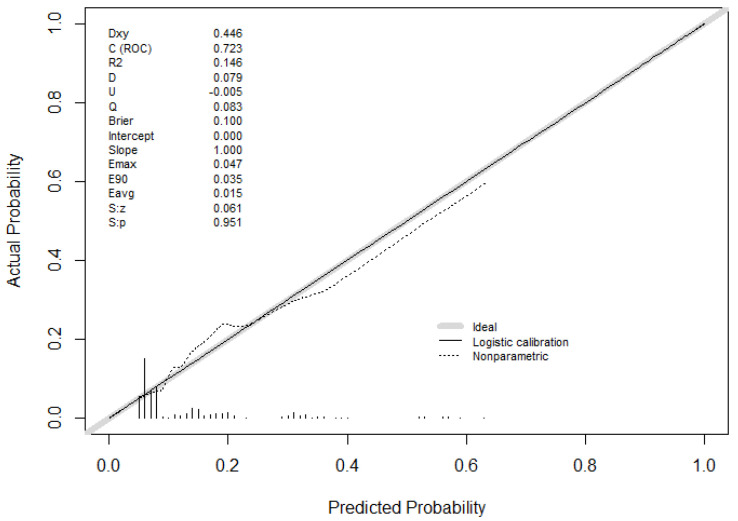
Calibration curve created using the val.prob function in R. The nonparametric line is a lowess calibration curve.

The whole model intercept estimate was −2.98. In order to evaluate how the predicted values generated by our model differ from the values after calibration, we used the val.prob function in R to generate a calibration curve and fit a nonparametric lowess line, showing the bias of our model compared to the nonparametric one (Figure 3). A Dxy 0.45 shows good correlation between actual and predicted probability. The shape of the nonparametric line suggests that our model is more optimistic at low probabilities and more pessimistic at high probabilities when compared to the lowess line.

**Figure 4:**
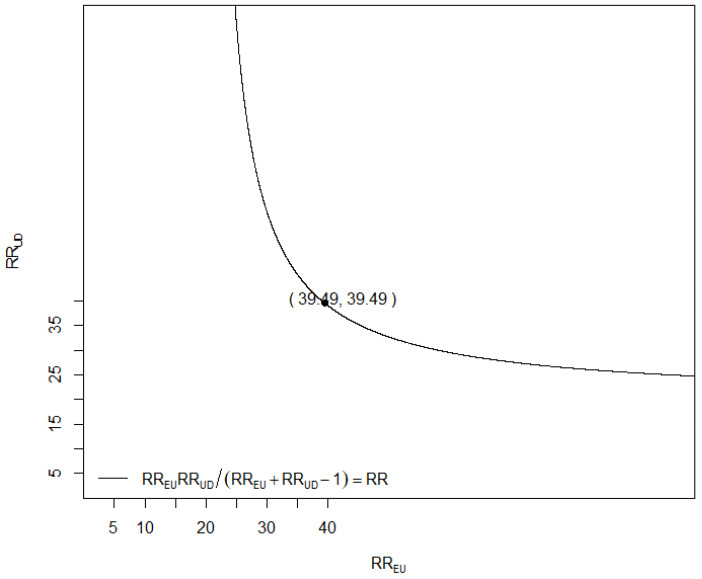
Plot showing the relationship needed between RR_EU_ and RR_UD_ to null our model–outcome relationship. As specified by VanderWeele et al. [16], ‘RR_EU_ = maximum risk ratio for any specific level of the unmeasured confounders comparing those with and without treatment, with adjustment already made for the measured covariates; RR_UD_ = maximum risk ratio for the outcome comparing any 2 categories of the unmeasured confounders, with adjustment already made for the measured covariates’.

We performed sensitivity analysis on our model using the EValue package in R. The E-value for the whole model odds ratio and confidence interval (specified in Table 3) was 39.43, meaning that a set of confounders would have to be associated with a 39.43 times increase in cardiac irAEs (RR_UD_) and must be 39.41 times more prevalent in people with cardiac irAEs (RR_EU_) to fully explain the relationship between our model and the outcome. We plotted how the strength of these relationships could vary in order to explain the association between our model and the outcome (Figure 4). A value of 39.41 shows little vulnerability to unmeasured confounding variables. Our outcome is rare, <15% at the end of follow-up, so the risk ratio (RR) could be approximated to our OR.

**Figure 5:**
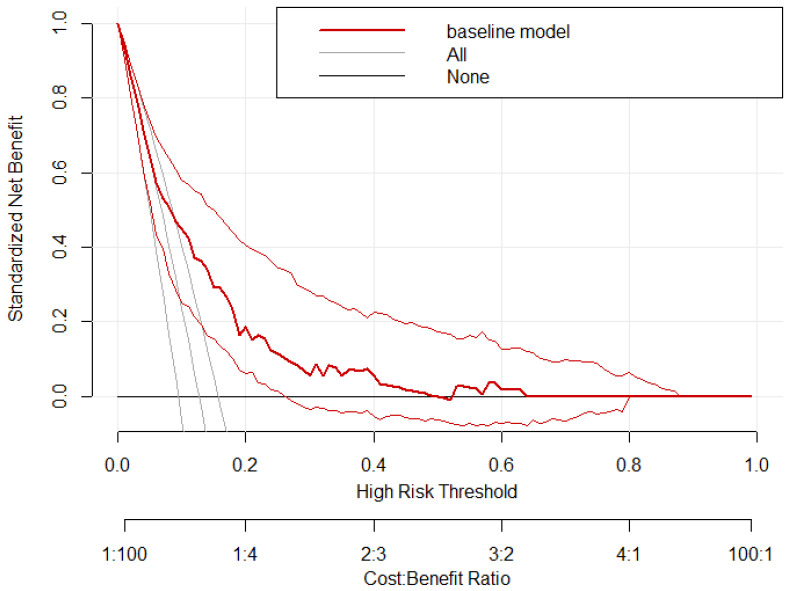
Decision curve made using the rmda package. All = all patients have cardiac irAEs receive treatment. None = no patient has cardiac irAEs and receives no treatment. Bold red line = main model, representing net benefit plotted against threshold probability. Light red lines = confidence intervals of the main model generated through empirical bootstrapping.

To briefly evaluate the clinical usefulness of our model, even though we did not intend to extract a prediction score, we calculated a decision curve using the rmda package (Figure 5). The y axis represents net benefit at certain thresholds, marked on the x axis. The None and All lines show that no patient has cardiac irAEs and receives no intervention and that all patients have cardiac irAEs and all receive intervention, respectively. Adherence to these lines show that at those thresholds the model is ineffective for clinical decision-making. However, at thresholds around 0.2–0.4 the model seems to have some benefit in discrimination and clinical decision-making.

### 3.3. Logistic Regression Models Predicting Non-Cardiac irAEs for the General Population

As a secondary objective, we used the same variables in order to see if any of them predict non-cardiac irAEs and assessed if there is any linkage between the effects. The only statistically significant predictors were prior radiation therapy, which showed a protective effect, and hypercholesterolemia, which was a risk factor for non-cardiac irAEs (Table 4). We did not fit a multiple logistic regression model because the selected predictors were not appropriate for non-cardiac irAEs.

### 3.4. Logistic Regression Models Predicting Cardiac irAEs for Lung Cancer and Head and Neck Cancer Patients

We selected our two most populous patient subgroups and applied the same model used at Section 3.2 to assess whether the predictors identified as statistically significant can be used in lung and head and neck cancer patients. For the former, only hypercholesterolemia was still a significant risk factor, while for the latter, having a history of acute coronary syndrome was also a risk factor. In the case of the head and neck subgroup, non-cardiac irAEs seem to predict cardiac irAEs (Table 5). These findings suggest that the overall effect shown at Section 3.2 is subtle and would require greater statistical power (a larger sample) to be detected. Odds ratios close to 9 and 10 should be interpreted critically as their values might be artificially inflated by the smaller subgroup size compared to the genera cohort.

## 4. Discussion

### 4.1. General Considerations

To our knowledge, this is the first logistic regression-based prediction model for pericardial disease, arrhythmias and myocarditis related to immune checkpoint inhibitors.

This study is an expansion of the previously published cohort study by our team, showing that cardiac irAEs are not associated with better outcomes [10]. We aimed to determine whether cardiovascular conditions and cardiovascular risk factors increase the risk of developing cardiac irAEs, consistent with the hypothesis that the development of cardiac irAEs is based on a different mechanism than that of other irAEs.

Our study shows that, based on our cohort, people with hypercholesterolemia, atrial fibrillation, hypertension, acute coronary syndrome, and cardiac failure prior to ICI therapy have an increased risk of developing cardiac irAEs when accounted separately. The statistical significance of this finding was maintained when including all of these predictors in a multiple regression model, except for increased blood pressure which lost its effect when accounting for the other covariates. There was no association between non-cardiac irAEs and cardiac irAEs.

When analyzing the ROC curve, we observe an acceptable AUC, indicating good discriminative power. The specificity of 0.98 paired with the low sensitivity shows that, according to the model based on our data, people with no cardiovascular condition prior to ICI therapy will not develop cardiac irAEs. While exciting, this finding should be further verified by external validation; as any model that emerged from one cohort, albeit large, it suffers from optimism bias and some overfitting.

In accordance with our line of thought, when presenting non-cardiac irAEs with the same set of predictors, only hypercholesterolemia was statistically significant.

Subgroup analyses of HN and lung cancer patients showed limited reproducibility of the results observed in the whole cohort. In the case of lung cancer, only hypercholesterolemia was a significant predictor, compared to HN cancers where non-cardiac irAEs and previous acute coronary syndrome were also significant risk factors. This situation might be caused by the simple logistic regressions being underpowered compared to the ones performed on the general population. There could also be a primary cancer location influence on the appearance of cardiac irAEs.

### 4.2. Influence of Cardiovascular Conditions and Cardiovascular Risk Factors on Developing Cardiac irAEs

In a meta-analysis published by Rubio-Infante et al. [17], evaluating data from 2015 to 2020, around 40% of people who developed cardiac irAEs had a cardiovascular risk factor, be it hypertension, hypercholesterolemia, atrial fibrillation, or coronary artery disease. More recent cohort or case–control studies tend to yield similar results. Chiang et al. performed a case–control study on around 800 patients in each arm, showing that, using a multiple logistic regression, hypertension was a risk factor for developing cardiac irAEs [18]. However, no other cardiovascular conditions were included in the models used to determine that. There was also no association between other irAEs and cardiac irAEs. A meta-analysis of 12 observational studies including 21,912 patients by Huang et al. showed that male gender, coronary artery disease, hypertension and chronic kidney disease were significant predictors for cardiovascular adverse events. The endpoint of this study was not identical to ours, encompassing a broader spectrum of cardiovascular adverse events, including de novo and worsening conditions. This may explain the associations between male gender and chronic kidney disease and the final outcome, these two variables being established cardiovascular disease risk factors [19].

Heilbroner et al. built a machine learning-based prediction model for cardiac irAEs, including new-onset heart failure, in addition to our endpoints. Their cohort had no head and neck patients and was far larger at 4960 patients. In their model, increased heart rate, decreased hemoglobin, decreased sodium and not receiving cardioprotective medication were associated with an increased risk of cardiac irAEs [20].

Power et al. built a multiple Cox regression model based on a composite outcome of severe arrhythmia, HF, respiratory muscle failure or cardiomyotoxicity-related death that included almost none of our predictors and performed well in external cohorts. It found that active thymoma, low QRS voltage on electrocardiogram, low left ventricular ejection fraction (<50%), cardiomuscular symptoms and troponin elevation were all risk factors for developing an event found in their composite index. Although we included ejection fraction in our model, we treated it as a continuous predictor rather than a binary (<50%) yes/no variable, which may explain why it was not significant in our study. This is consistent with the low sensitivity of our model, suggesting that several other predictors may explain the occurrence of cardiac irAEs. Another major difference lays in the datasets, a particularity of our cohort being its increased number of head and neck cancer patients [21].

Oren et al., in a cohort of around 3000 patients, built a prediction model for ICI myocarditis, based on Cox regressions. Similarly to our data, history of acute coronary syndrome and heart failure were significant predictors. In this case, hypercholesterolemia was positively associated with decreased mortality [22].

In a systematic review of case reports and case series, including 223 cases of immunotherapy-induced myocarditis, Nielsen et al. found that 16.3% had diabetes, 41.1% had hypertension and 46.5% had other cardiovascular risk factors, showing that almost half of the patients had some form of prior cardiovascular stress. A total of 91% of the patients were older than 60; while age was not a statistically significant predictor in our model, it almost reached significance with a *p* value of 0.55 [23].

Isawa et al. showed, using Fine-Gray multivariate regression on a single-center NSCLC retrospective cohort, that prior acute coronary syndrome and a history of hospitalization for heart failure were associated with cardiovascular irAEs [24]. While there are other Romanian studies focused on NSCLC and immunotherapy, they do not follow cardiotoxicities; our subgroup is also more heterogenous, including both SCLC and NSCLC patients [25,26].

Haj-Yehia et al. found in a prospective cohort of patients with pre-existing cardiac disease that increased neutrophil-to-lymphocyte (NLR) ratio is associated with cardiovascular toxicity, myocarditis and arrythmia or QTc prolongation [27]. While increased NLR is an established negative prognostic marker in both oncological and cardiovascular disease [28,29], decreased baseline values are associated with the development of overall irAEs in some cohorts [30]. An interesting study by Matsukane et al. found that, when continuously monitored, NLR increases during the development of irAEs. After the irAE passes, there are two categories of patients: the ones that return to the baseline NLR value, showing better PFS, and the ones who maintained elevated NLR, showing worse PFS [31].

When analyzed by tumor type, there is no definitive evidence associating the localization of malignancy with the development of cardiac irAEs. While melanoma and non-small cell lung cancer seem to be associated with an increased rate of cardiovascular irAEs, this could be a result of overreporting, caused by increased use of ICIs in those localizations [32]. Our cohort is particularly different through the increased number of head and neck patients; while not that frequent in the general population, Reyes-Gibby et al. found that an incidence of approximately 35% cardiovascular events in their cohort comprised 610 head and neck patients [33]. Cardiovascular irAEs were also extensively reported in urothelial cancer, renal cancer, and hepatocellular cancer, while there were some cases found in the treatment of mesothelioma [34] or cholangiocarcinoma [35,36].

### 4.3. Explanation of the Mechanisms of Cardiac irAEs and the Relationship Between These and Our Logistic Regression Model

In our cohort there were no statistically significant associations between non-cardiac irAEs and cardiac irAEs; no cardiovascular factor except for hypercholesterolemia was significant in predicting non-cardiac irAEs. In the previous study published on this cohort, we found that cardiac irAEs had no benefit on survival, while non-cardiac irAEs had significant benefit [10]. This pattern seems to repeat in other observational studies in the literature [37,38,39].

Considering the results from our logistic regression model, we launch the following mechanistic hypothesis regarding cardiac irAEs: the occurrence of cardiac irAEs is based on an already primed cardiac terrain; however, this is not the only requirement. In the case of our cohort, the high specificity, coupled with the low sensitivity and low model fit, suggests that developing a cardiac irAE in the form of de novo arrythmia, myocarditis or pericardial disease almost always requires some form of cardiovascular predisposition. However, having such a predisposition while being a prerequisite, it does not equal developing a cardiac irAE. There are probably more mechanisms at bay that have a protective effect.

A 2025 ACC statement established the role of inflammation in cardiovascular disease. ACC now recommends using interleukin-6 and high sensitivity C reactive protein as risk predictors in heart failure and declares inflammation as a pivotal process in atherosclerosis and plaque progression [40]. In the spirit of this statement, hypercholesterolemia was a significant risk factor for both cardiac and non-cardiac irAEs in our study, underscoring the interplay between a marker of atherosclerosis and a pro-inflammatory state.

The role of the PD-1/PD-L1 pathway in cardiovascular disease has been extensively discussed by Sun et al. They highlight several murine models that correlate atherosclerosis and PD-1/PD-L1 deficiency. They cite two studies on human peripheral blood cells: the first by Lee et al., which correlates low PD-1 expression on T cells with coronary artery disease, and the second by Li et al., which correlates high PD-L1 expression on Tregs with acute coronary syndrome and chronic heart disease [41,42,43]. Inflammatory cytokines such as interferon-γ seem to induce the expression of PD-L1 on cardiac tissue in an upregulation fashion. Experimental models of ex vivo mice with ischemic–reperfusion lesions show increased expression of PD-L1 on cardiac cells. This is believed to be a pathway of myocardial protection from acute inflammatory injury [44,45]. Another murine model by Hayashi et al. showed that isoproterenol-induced stress cardiomyopathy upregulates PD-1/PD-L1 in resident heart lymphocytes; furthermore, a 40% increase in mortality was observed in mice treated with checkpoint inhibitors [46]. Choudhary et al. performed a study on endomyocardial biopsies from 19 heart transplant patients and found that myocardial PD-L1 is upregulated by acute cellular rejection in accordance with its severity [47]. Kushnareva et al. [48] analyzed 12 autopsy samples from patients with myocardial infarction, using a control group of 10 patients with cancer that received no treatment and 4 patients with cancer that received ICI. The results were very interesting: only the experimental group and patients treated with ICI who had a history of cardiovascular disease expressed membrane, endothelial and intercalated disks PD-L1. These patients showed a more pronounced cytoplasmic PD-L1 expression.

Although the myocardial implications of the PD-1/PD-L1 axis are not completely understood and need further in vivo and human research, there is growing evidence showing upregulation in heart injury and inflammation. This steady state created through this protective mechanism seems to be interrupted by ICI therapy, inducing irAEs in patients with prior cardiovascular disease [49].

## 5. Conclusions

This study provides evidence that pre-existing cardiovascular comorbidities play a significant role in the development of immune-related cardiac adverse events in patients treated with immune checkpoint inhibitors. Hypercholesterolemia, atrial fibrillation, prior acute coronary syndrome, and heart failure were identified as the main factors associated with an increased risk of cardiac irAEs, supporting the hypothesis that cardiac toxicity occurs preferentially in a previously vulnerable cardiovascular setting.

The predictive model demonstrated high specificity, suggesting that patients without pre-existing cardiovascular disease are unlikely to develop immune-mediated cardiac toxicity. However, the modest sensitivity indicates that additional biological or clinical factors may contribute to the pathogenesis of cardiac irAEs and remain to be identified. Notably, non-cardiac irAEs were not associated with cardiac toxicity, supporting the concept that cardiac irAEs represent a distinct clinical entity rather than part of a generalized immune activation pattern.

These findings underscore the importance of careful cardiovascular assessment prior to initiation of immunotherapy and support the need for tailored monitoring strategies in patients with known cardiovascular comorbidities. Further prospective studies and external validation cohorts are warranted to refine risk-stratification models and elucidate the mechanisms underlying immune-related cardiotoxicity.

## Figures and Tables

**Table 1:** Additional data collected for the objective of this study. Cardiac comorbidities and risk factors of our patients prior to immune checkpoint inhibitors therapy.

Variable		Number	Frequency (%)
Chronic Kidney Disease *	Yes	44	10.7
	No	366	89.3
Atrial Fibrillation **	Yes	34	7.9
	No	399	92.1
Acute Coronary Syndrome	Yes	30	6.9
	No	405	93.1
Heart failure	Yes	53	12.2
	No	382	87.8

* 25 patients missing data, ** 2 patients missing data.

**Table 2:** Simple logistic regression models predicting cardiac irAEs for the general population.

Variable	Odds Ratio (95% CI)	*p*-Value
**General predictors**		
Age	1.029 (0.999 to 1.059)	0.055
Prior radiotherapy	0.789 (0.448 to 1.389)	0.67
Prior chemotherapy	1.136 (0.638 to 2.022)	0.66
Non-cardiac irAEs	1.384 (0.728 to 2.631)	0.32
Cardiac-related predictors		
Diabetes (Yes)	1.486 (0.769 to 2.873)	0.24
Chronic kidney disease (Yes)	1.546 (0.677 to 3.531)	0.3
**Hypercholesterolemia (Yes)**	**3.023 (1.67 to 5.472)**	**<0.001**
**Atrial fibrillation (Yes)**	**3.859 (1.762 to 8.45)**	**<0.001**
**Hypertension (No)**	**0.485 (0.269 to 0.876)**	**0.016**
**Acute coronary syndrome (Yes)**	**4 (1.762 to 9.081)**	**<0.001**
Ejection fraction	0.982 (0.928 to 1.04)	0.54
**Heart failure**	**4.275 (2.194 to 8.329)**	**<0.001**

**Table 3:** Multiple logistic regression model predicting cardiac irAEs for the general population.

Variable	Odds Ratio (95% CI)	*p*-Value	McFadden R^2^
Whole model (Intercept)	0.146 (0.006 to 0.421)	**<0.001**	0.106
Age	1.009 (0.977 to 1.041)	0.602	
Hypercholesterolemia (Yes)	**1.954 (0.999 to 3.822)**	**0.05**	
Atrial fibrillation (Yes)	**2.755 (1.185 to 6.404)**	**0.019**	
Hypertension (No)	0.826 (0.422 to 1.616)	0.58	
Acute coronary syndrome (Yes)	**2.763 (1.144 to 6.675)**	**0.024**	
Heart failure (Yes)	**2.687 (1.296 to 5.571)**	**0.008**	

**Table 4:** Simple logistic regression models predicting non-cardiac irAEs for the general population.

Variable	Odds Ratio (95% CI)	*p*-Value
**General predictors**		
Age	1.014 (0.992 to 1.037)	0.22
Prior radiotherapy (Yes)	**0.581 (0.368 to 0.918)**	**0.02**
Prior chemotherapy (Yes)	1.479 (0.924 to 2.367)	0.1
Cardiac irAEs	1.384 (0.728 to 2.631)	0.33
Cardiac-related predictors		
Diabetes (Yes)	1.302 (0.752 to 2.255)	0.35
Chronic kidney disease (Yes)	0.77 (0.344 to 1.721)	0.52
Hypercholesterolemia (Yes)	**1.757 (1.054 to 2.930)**	**0.031**
Atrial fibrillation (Yes)	1.563 (0.719 to 3.395)	0.26
Hypertension (No)	0.69 (0.437 to 1.09)	0.11
Acute coronary syndrome (Yes)	1.081 (0.449 to 2.6)	0.86
Ejection fraction	0.959 (0.913 to 1.007)	0.096
Heart failure	1.038 (0.522 to 2.065)	0.107

**Table 5:** Simple logistic regression models predicting cardiac irAEs for lung and head and neck cancer patients (only statistically significant predictors are shown).

Variable	Odds Ratio (95% CI)	*p*-Value
**Lung cancer**		
Hypercholesterolemia (Yes)	2.447 (1.187 to 5.041)	0.015
**Head and neck cancer**		
Hypercholesterolemia (Yes)	10.083 (1.95 to 52.137)	0.006
Acute coronary syndrome	10 (1.545 to 64.729)	0.016
Non-cardiac irAEs	9.5 (1.946 to 46.388)	0.005

## Data Availability

Data available on request due to ethical restrictions. The data presented in this study are available on request from the corresponding author and the Coltea Clinical Hospital (secretariat@coltea.ro). The data are not publicly available due to the policy of Coltea Clinical Hospital requiring the approval of the Ethics Committee for each new research study.

## Appendix A

**Table A1:** Demographic and clinical characteristics of the patients included in the study. Reproduced from Pătru et al. [10].

Variable		Number	Frequency (%)
Sex	Male	313	72.0
	Female	122	28.0
ECOG	ECOG 0	137	31.5
	ECOG 1	124	28,5
	ECOG 2	167	38.4
	ECOG 3	7	1.6
Smoking status *	Smoker	234	53.9
	No smoker	200	46.1
DM prior to ICIs	Yes	85	19.5
	No	350	80.5
HT prior to ICIs	Yes	218	50.1
	No	217	49.9
Hypercholesterolemia prior to ICIs	Yes	96	22.1
	No	339	77.9
Diagnosis	Lung cancer	219	50.3
	Head and neck cancer	137	31.5
	Melanoma	43	9.9
	Renal cell carcinoma	16	3.7
	Urothelial carcinoma	12	2.8
	Hepatocellular carcinoma	4	0.9
	Breast cancer	2	0.5
	Cancer of Unknown Primary	2	0.5
CHT prior to ICIs **	Yes	184	42.3
	No	247	56.8
RT prior to ICIs	Yes	236	54.3
	No	199	45.7
ICIs	Atezolizumab	20	4.6
	Avelumab	7	1.6
	Durvalumab	12	2.8
	Nivolumab	140	32.2
	Nivolumab + Ipilimumab	22	5.1
	Pembrolizumab	234	53.8

* One patient has missing data. ** 4 patients have missing data on previous chemotherapy. CHT—systemic chemotherapy, DM—diabetes mellitus, HT—hypertension, RT—radiation therapy.

**Table A2:** Cardiovascular events of the patients included in the study. Reproduced from Pătru et al. [10].

Cardiovascular events (n = 144, 33.1%)	Cardiomyopathy	45	10.3
	Hypertension	20	4.6
	Vascular toxicity (thrombosis)	55	12.6
Cardiac irAEs (n = 55, 12.6%)	Arrhythmias	35	8
	Grade 2	19	4.4
	Grade 3	5	1.1
	Grade 1	10	2.3
	Grade 4	1	0.2
	Pericardial disease	15	3.4
	Grade 2	6	1.4
	Grade 3	6	1.4
	Grade 1	2	0.5
	Grade 4	1	0.2
	Myocarditis	13	3
	Grade 2	8	1.8
	Grade 3	4	0.9
	Grade 4	1	0.2
